# Controlled Coprecipitation of Amorphous Cerium-Based Carbonates with Suitable Morphology as Precursors of Ceramic Electrolytes for IT-SOFCs

**DOI:** 10.3390/ma12050702

**Published:** 2019-02-27

**Authors:** Grazia Accardo, Gianfranco Dell’Agli, Maria Cristina Mascolo, Luca Spiridigliozzi, Sung Pil Yoon

**Affiliations:** 1 Center of Hydrogen-Fuel Cell Research, Korea Institute of Science and Technology, Hwarangno 14-gil, Seongbuk-gu, Seoul 136-791, Korea; d16605@kist.re.kr (G.A.); spyoon@kist.re.kr (S.P.Y.); 2 Department of Civil and Mechanical Engineering, University of Cassino and Southern Lazio, Via G. Di Biasio 43, 03043 Cassino (FR), Italy; mc.mascolo@unicas.it (M.C.M.); l.spiridigliozzi@unicas.it (L.S.)

**Keywords:** amorphous coprecipitate, ammonium carbonate, doped ceria, hydroxycarbonates, sintering

## Abstract

To be suitable as electrolytes in intermediate temperature solid oxide fuel cell (IT-SOFC), ceramic precursors have to be characterized by high sintering aptitude for producing fully densified products which are needed for this kind of application. Therefore, synthesis processes able to prepare highly reactive powders with low costs are noteworthy to be highlighted. It has been shown that amorphous coprecipitates based on cerium doped (and codoped) hydrated hydroxycarbonates can lead to synthesized ceramics with such desired characteristics. These materials can be prepared by adopting a simple coprecipitation technique using ammonium carbonate as precipitating agent. As a function of both the molar ratio between carbonate anions and total metallic cations, and the adopted mixing speed, the coprecipitate can be either amorphous, owning a very good morphology, or crystalline, owning worse morphology, packing aptitude, and sinterability. The amorphous powders, upon a mild calcination step, gave rise to the formation of stable solid solutions of fluorite-structured ceria maintaining the same morphology of the starting powders. Such calcined powders are excellent precursors for sintering ceramic electrolytes at low temperatures and with very high electrical conductivity in the intermediate temperature range (i.e., 500–700 °C). Therefore, irrespective of the actual composition of ceria-based systems, by providing an accurate control of both chemical conditions and physical parameters, the coprecipitation in the presence of ammonium carbonate can be considered as one of the most promising synthesis route in terms of cost/effectiveness to prepare excellent ceramic precursors for the next generation of IT-SOFC solid electrolytes.

## 1. Introduction

In the last few decades, ceria (CeO_2_)-based ceramics have attracted much interest as functional materials in many applications such as chemical-mechanical polishing media, automobile exhaust catalysts and supports, dense ceramic membranes for oxygen separation, low temperature water-gas shift (WGS) reaction catalysts, oxygen sensors, etc. [1,2]. However, the readiest application for a real market stage exploitation of ceria-based ceramics is related to the production of solid electrolytes for intermediate temperature solid oxide fuel cells (IT-SOFCs), due to their very high ionic conductivity and slow degradation under operating conditions (500–700 °C, i.e., the typical temperature range of the IT-SOFCs) [3,4].

The reduction of SOFCs operating temperature is strongly required for efficient long-term operations and this reduction can be fulfilled by using a new class of ceramic electrolytes (i.e., ceria-based) as substitute of the commonly used Y_2_O_3_-doped ZrO_2_ (YSZ) [5]. Furthermore, the powder precursors of these materials should be produced through a simple and cheap process, easily scalable at industrial level and characterized by a very high yield, thus making IT-SOFCs commercial exploitation actually real [6]. Several classes of materials have been proposed in literature as alternative to YSZ ceramic electrolyte for IT-SOFCs such as bismuth-based oxides, zirconia-based oxides [7], ceria-based oxides [8], and lanthanum gallate-based oxides [9], but among them, the most widely studied are doped/codoped ceria and, in particular, Sm-doped CeO_2_ [10], Gd-doped CeO_2_ [11], and Gd/Sm codoped CeO_2_ [12,13,14].

Unfortunately, ceria-based ceramics prepared by conventional solid-state synthesis are characterized by a poor sintering aptitude, thus requiring high temperatures, long dwell times, and low heating rates to produce sufficiently densified ceramic bodies [15]. A first step to overcome these drawbacks consists in the use of sintering aids [16,17], whereas a more effective way to do it relies on the preparation of very reactive powders which are capable to be sintered at significantly reduced temperatures. Many synthesis routes fulfill this requirement and, among them, the most common ones are the hydrothermal treatment [18,19], the sol–gel route [20] and the coprecipitation method from aqueous solutions [4,21]. Moreover, among those processes, the coprecipitation method is the simplest and cheapest one, even if powders prepared via coprecipitation from aqueous solutions are often characterized by high degrees of agglomeration causing the formation of hard aggregates during the calcination and sintering steps, thus consequently impairing severely the final density of the electrolyte [22,23]. Finally, the either amorphous or crystalline nature of the precipitated powders can dramatically influence their morphology and, in turn, their forming aptitude and sinterability.

In a very recent work [4], we revealed that the use of ammonium carbonate as precipitating agent allowed the preparation of amorphous precursors of softly agglomerated 20% mol Sm-doped ceria powders with a quite ideal morphology, thus having an excellent sintering behavior. Therefore, based on these results, the aim of this work was to verify if by using our engineered synthesis method also to several more complex doped and codoped (with both rare-earth, e.g., Pr, and earth-alkaline metals, e.g., Ca) ceria-based systems, amorphous powders with such quite ideal morphology can be obtained; also these amorphous powders are useful precursors of ceria-based ceramics with excellent sintering behavior and electrochemical properties. In particular, the influence of different doping elements and the effect of chemical-physical synthesis parameters have been highlighted.

## 2. Materials and Methods

Cerium(III) nitrate (Ce(NO_3_)_3_·6H_2_O, 99.0% Sigma-Aldrich, Milan, Italy), samarium(III) nitrate (Sm(NO_3_)_3_·6H_2_O, 99.9% Sigma-Aldrich, Milan, Italy), gadolinium(III) nitrate (Gd(NO_3_)_3_·6H_2_O, 99.0% pure, Sigma-Aldrich, Milan, Italy), praseodymium(III) nitrate (Pr(NO_3_)_3_·6H_2_O, 99.0% Sigma-Aldrich, Milan, Italy), calcium nitrate (Ca(NO_3_)_2_·4H_2_O, 99.0% Carlo Erba, Cornaredo, Italy), and ammonium carbonate ((NH_4_)_2_CO_3_, 99.0% Fluka-Honeywell, Sigma-Aldrich, Steinheim, Germany) were used as reagents for the synthesis of the various samples. All the six (6) synthesized samples, their coprecipitation conditions, and labeling are reported in Table 1.

Regardless of sample composition, the adopted synthesis procedure for the first five samples in Table 1, e.g., the ones prepared with fast mixing of precursors, was the same as described hereafter. Firstly, the proper amount of rare earth (and alkaline earth for SCaDC20) nitrates was dissolved in deionized water until a total cationic concentration of 0.1 M has been reached (solution A). Subsequently, an aqueous solution of ammonium carbonate (0.5 M) was prepared (solution B). The two solutions were vigorously stirred for 1 h to obtain full dissolution and homogenization. The coprecipitation was carried out at room temperature by quickly adding (fast mixing in Table 1 and in the following) solution B to solution A, keeping the resulting suspension under vigorous stirring and adjusting the volumes’ ratio to R = 2.5, R being the molar ratio between carbonate anions and total metal cations. This selected R value corresponds to a slight excess of carbonate and it is derived from previous literature evidences [4,24]. When the two solutions are mixed, a solid coprecipitate is instantly formed and subsequently recovered by vacuum filtration. The so-obtained powders were then repeatedly (five times, with the same standardized procedure) washed with deionized water and finally dried overnight at 80 °C. Instead, the sample SDC20R10S was prepared by slowly adding (slow mixing in Table 1 and in the following) solution B to solution A (≈ 10 mL/min) to allow coprecipitates crystallization, and keeping the molar ratio R = 10.

All the as-synthesized powders were calcined at 600 °C for 1.5 h under static air atmosphere to induce the thermal decomposition of the as-formed phases and to allow the complete crystallization of the desired fluorite-structured doped ceria. The calcined powders were subsequently compacted into cylindrical pellets by uniaxial pressing and then sintered in air at 1300 °C for 3 h to obtain the dense electrolytes for characterization. A heating rate of 5 °C min^−1^ was always used.

All the synthesized powders were characterized by X-ray powder diffraction (XRD) by using a diffractometer (X’PERT, Panalytical, Almelo, The Netherlands) with CuKα radiation. The crystal size of the samples was calculated by the Scherrer formula, whereas the cell parameter of fluorite phases were calculated by the least-square procedure reported by Razik [25].

The thermal behavior of the powders was determined with thermogravimetric analysis and differential thermal analysis (TGA-DTA) in air (STA 409 Thermoanalyzer, Netzsch Instruments, Selb, Germany) with heating rate of 10 °C∙min^−1^ up to 1200 °C and α-Al_2_O_3_ as a reference. Powder morphology and sintered pellets microstructure was highlighted by Scanning Electron Microscopy (SEM), using an instrument (Novasem, FEI Co., Hillsboro, OR, USA) equipped with both the standard Everhart-Thornley detector (ETD) and a through lens detector (TLD to better highlight crystal surfaces at higher magnifications. Before collecting SEM micrographs, powders were coated with a thin layer of conducting material with a sputtering deposition process (E-1045 Ion sputter, Hitachi, Tokyo, Japan). The vacuum degree during platinum coating was 6 Pa, while the discharge current was fixed at 15 mA for 120 sec. SEM images instead were detected at 50000X of magnification at spot size of 3.5 and acceleration voltage of 20 kV.

The sintering behavior of the various samples was investigated by thermodilatometry tests carried out in air using a DIL 402PC dilatometer (Netzch Instruments, Selb, Germany) with 5 °C·min^−1^ as heating rate up to 1400 °C. 

The electrical behavior of dense electrolytes was investigated by Electrochemical Impedance Spectroscopy (EIS) in air. A platinum conductive paste was painted on both pellets sides as electrodes. EIS was carried out in the 300 to 800 °C range by using a frequency response analyzer (FRA, Solartron 1260, Ametek, Leicester, UK) coupled with a dielectric interface (Solartron 1296, Ametek, Leicester, UK) in the frequency range of 0.1 Hz to 1 MHz applying an AC voltage amplitude of 100 mV. Data analysis of measured impedances was carried out by using ZPlot and Zview software (Scribner Associates Inc, Southern Pines, NC, USA).

## 3. Results and Discussion

**Figure 1:**
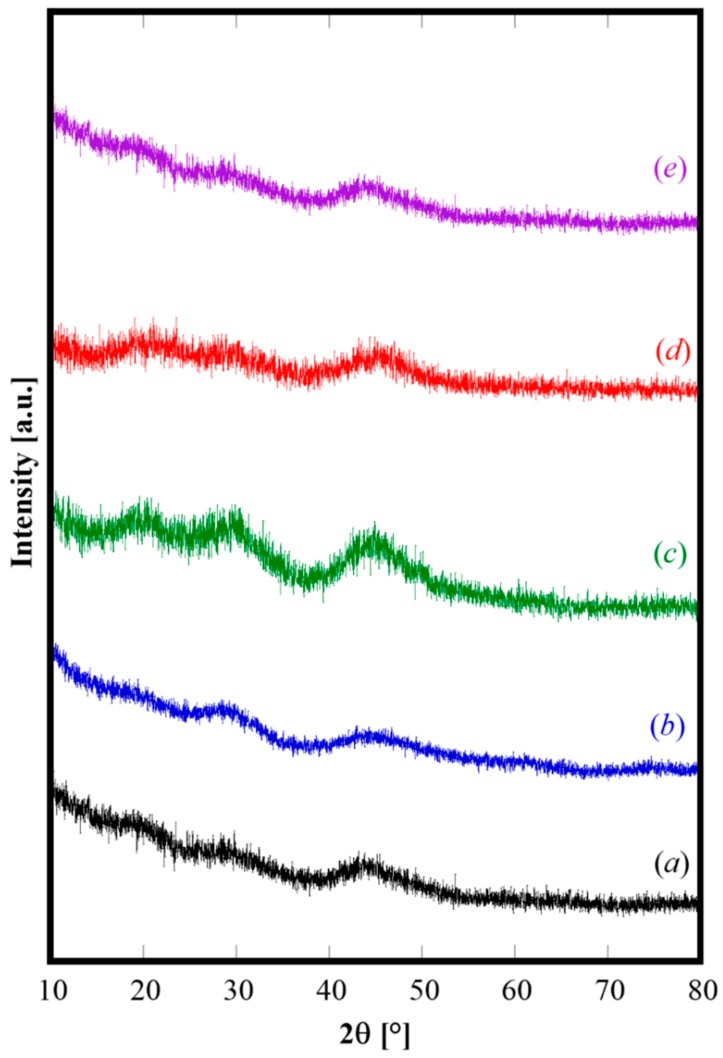
X-ray powder diffraction (XRD) patterns of samples C (**a**), GDC20 (**b**), SDC20 (**c**), GPrDC20 (**d**), and SCaDC20 (**e**).

Irrespective of the actual composition of solution A, the coprecipitates obtained with R = 2.5 and fast mixing are all amorphous. In fact, the XRD patterns of the samples prepared with the fast mixing procedure (see Table 1), displayed in Figure 1, exhibit all only some diffraction halo. 

**Figure 2:**
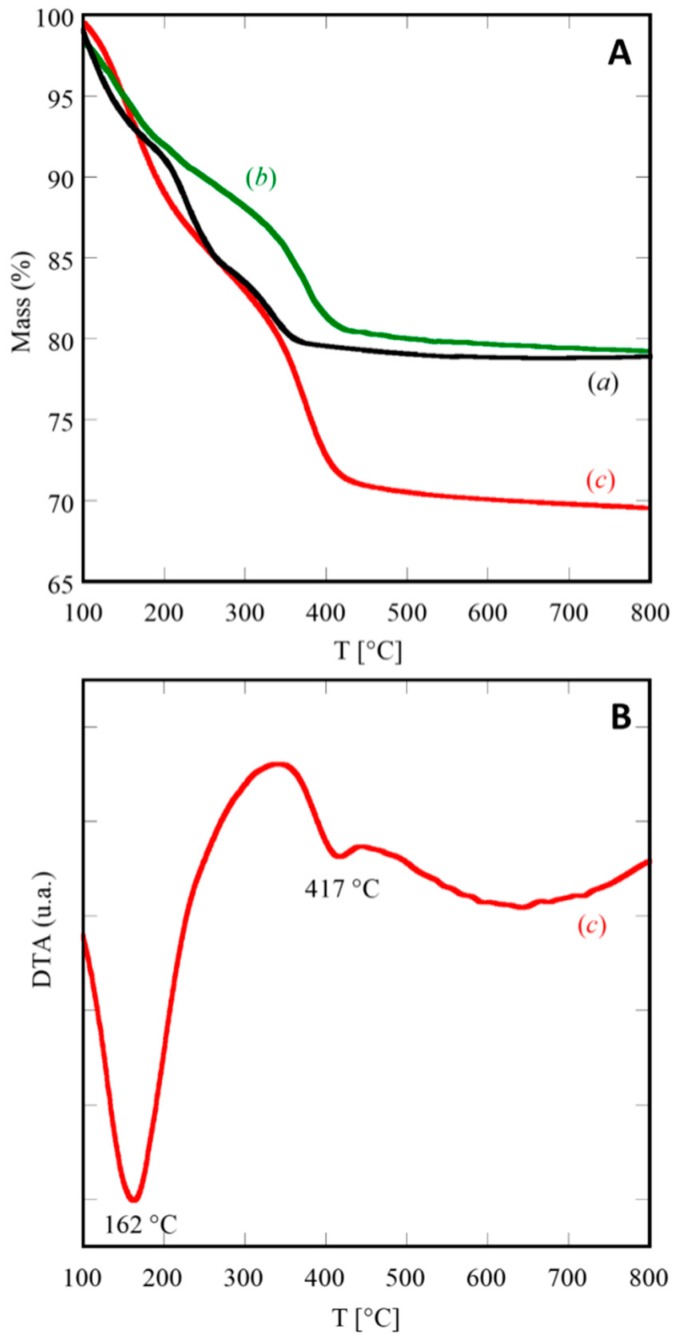
(**A**) Thermogravimetric analysis (TGA) curves of some selected samples: C (a), SDC20 (b), and GPrDC20 (c). (**B**) Differential thermal analysis (DTA) curve of sample GPrDC20.

Obviously, the phases present in the amorphous coprecipitates are not directly detectable. Based on both literature reports [4,24] and on the related thermogravimetric analysis (See Figure 2), it can be deduced that the obtained amorphous phase could be a cerium(III) hydrated hydroxycarbonate wherein, depending on the actual composition of different samples, some Ce^3+^ cations are replaced by the dopant metals. In fact, indicating with RE cerium or the mixture of cerium and other rare-earth dopant cations (i.e., Gd, Sm, or Pr), the possible route forming the amorphous coprecipitate, as also suggesting by Hirano [26], is a two-step process: the first one, i.e., Equation (1), is the typical hydrolysis reaction of RE^3+^ cations in aqueous solution and the second one, i.e., Equation (2), is a precipitation reaction leading to the formation of insoluble amorphous rare earth hydroxycarbonate:

(1) $${\left[RE{\left(H_{2}O\right)}_{n}\right]}^{3+}+H_{2}O\to {\left[REOH{\left(H_{2}O\right)}_{n-1}\right]}^{2+}+H_{3}O^{+}$$

(2) $${\left[REOH{\left(H_{2}O\right)}_{n-1}\right]}^{2+}+CO_{3}^{2-}\to amorphous-RECO_{3}OH\cdot xH_{2}O+\left(n-1-x\right)H_{2}O$$

where the x-value is influenced by the actual composition of RE^3+^ cation, as also reported by Moscardini D’Assuncao et al. [27], but very probably also by the drying step, so that differently hydrated hydroxycarbonates can be obtained. In fact, when the drying step is not controlled or monitored, the product properties may differ, and powders could not be suitable for electrolyte sintering. This aspect is currently under investigation and not further addressed in this work.

As a confirmation of XRD results, the thermographs in Figure 2 suggest that the transition from the amorphous coprecipitate to fluorite-type ceria-based product occurs always below 500 °C.

The TGA curves reported in Figure 2A display the thermal behaviors occurring during the heating up to 800 °C for three selected samples: C, SDC20, and GPrDC20. The resulting total weight losses are approximately 21% for the first two ones (see Figure 2A(a),(b)) and approximately 31% for the last one. These values allow to hypothesize that x = 0 when RE = Ce and RE = Sm_0.20_Ce_0.80_, whereas x = 2 for RE = Gd_0.16_Pr_0.04_Ce_0.80_. The thermal decomposition of these samples does not occur in a single step, as it is well-known for rare-earth hydroxycarbonates [28].

For instance, the decomposition of (Gd,Pr)-doped cerium(III) hydroxycarbonate, relative to sample GPrDC20, occurs in two distinct steps, well highlighted in the corresponding DTA plot, shown in Figure 2B, and marked by the two endothermic peaks at 162 °C and 417 °C. A possible decomposition route of the amorphous precursor of GPrDC20, whose chemical formula based on the above discussion is (Gd_0.16_Pr_0.04_Ce_0.80_)CO_3_OH·2H_2_O, could be represented by the following global chemical reactions.

(3) $$2\left(Gd_{0.16}Pr_{0.04}Ce_{0.8}\right)CO_{3}OH\cdot 2H_{2}O\to {\left(Gd_{0.16}Pr_{0.04}Ce_{0.8}\right)}_{2}O{\left(CO_{3}\right)}_{2}+5H_{2}O↑$$

(4) $${\left(Gd_{0.16}Pr_{0.04}Ce_{0.8}\right)}_{2}O{\left(CO_{3}\right)}_{2}+0.4O_{2}\to 2\left(Gd_{0.16}Pr_{0.04}Ce_{0.8}\right)O_{1.9}+2CO_{2}↑$$

Equation (3) and Equation (4) correspond to the two decomposition steps in Figure 2A(c). The weight losses associated with these two steps are approximately equal, representing an indirect confirmation of the proposed thermal decomposition model, as the amount of released H_2_O (Equation (3)) and CO_2_ (Equation (4)) are practically the same (i.e., 17.6 mg of H_2_O vs 17.2 mg of CO_2_ per gram of (Gd_0.16_Pr_0.04_Ce_0.80_)CO_3_OH·2H_2_O). Furthermore, it is interesting to notice from the DTA plot (Figure 2B) that the endothermic peak (at 417 °C), associated with the decarbonation (Equation (4)), is much smaller if compared with the other one. Very likely, as the evolution of CO_2_, the oxidation of Ce^3+^ to Ce^4+^, and the crystallization of fluorite-type ceria occurred simultaneously (or, better, in the same temperature range), the exothermicity of the crystallization of fluorite structure partly counterbalances the endothermicity of the evolution of CO_2_. Finally, the thermal stability of RECO_3_OH revealed by the DTA-TGA measurements is ensured in a lower temperature range than that reported by Kim [28] and referred to RE with fixed valency. In our opinion, the oxidation of Ce^3+^ to Ce^4+^ during the heating in the DTA-TGA run, heavily influences the thermal stability of the various possible phases favoring the breakdown of the carbonate structures and expediting the achievement of equilibrium.

Therefore, a mild calcination step at 600 °C for 1.5 h was selected for completing all the thermal events and allowing the formation of fluorite-structured doped/codoped ceria. Furthermore, these mild conditions prevent some undesired phenomena such as excessive grain growth and consequent reduction of powders reactivity.

**Figure 3:**
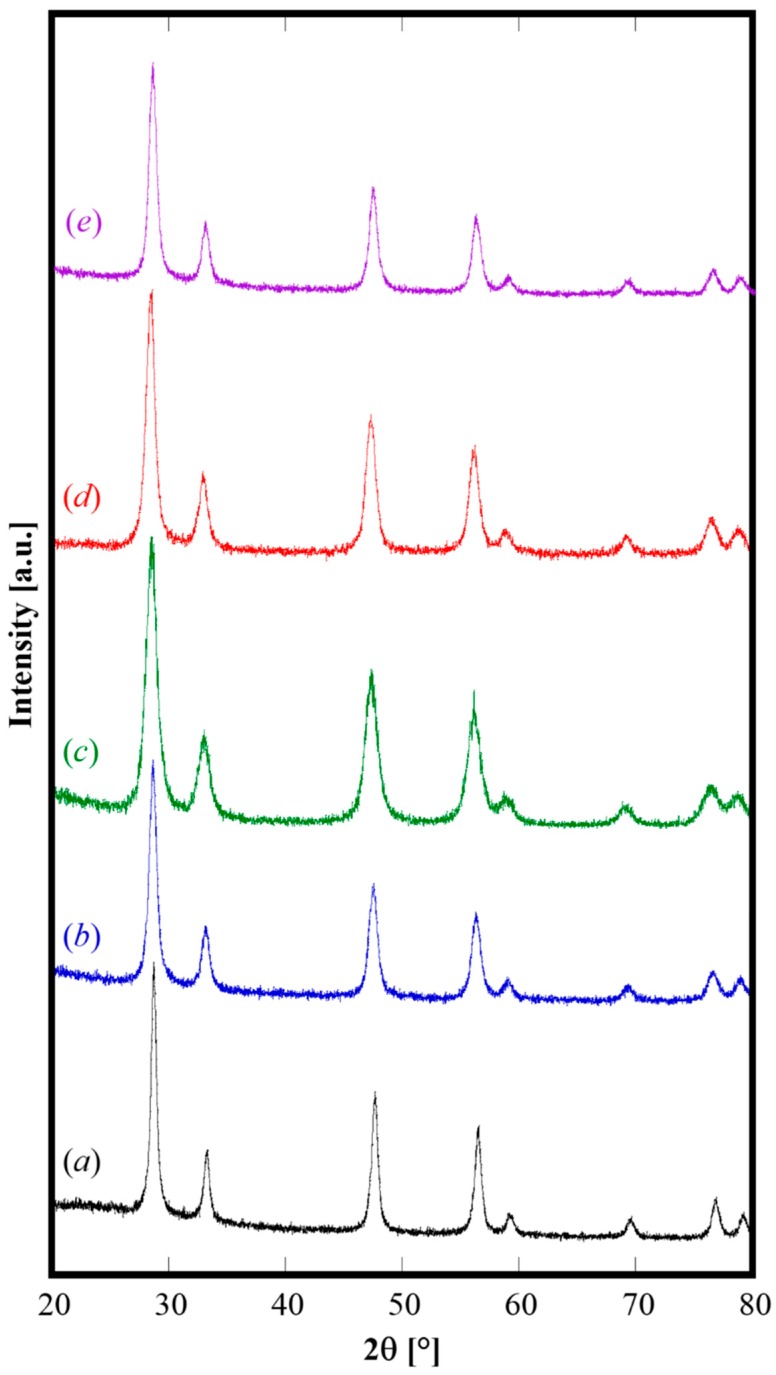
XRD patterns of calcined samples C (**a**), GDC20 (**b**), SDC20 (**c**), GPrDC20 (**d**), and SCaDC20 (**e**).

The XRD patterns of all the calcined samples, displayed in Figure 3, are all nearly identical except for a small shift in the peak positions. These samples are formed only by the fluorite-like phase, and the corresponding crystallographic parameters are reported in Table 2. The lattice parameters are all well consistent with the literature data for materials with analogous composition. Regarding the sample containing Pr as codopant, because of its well-known redox behavior when it is dissolved in fluorite structures, the lattice parameter varies according to the ratio Pr^3+^ (r = 0.1126 nm) over Pr^4+^ (r = 0.096 nm). Based on lattice parameter calculated for sample GPrDC20 (see Table 2), it can be estimated that a large amount (~ 90%) of Pr is present as Pr^3+^ by using the procedure reported by Nicholas [29] for computing the lattice parameter for fluorite structure. Finally, the values of crystallite size in Table 2, lying in the range of 10 to 15 nm for all the samples, confirm that the mild calcination step preserved the nanometric character of the initially amorphous samples. 

**Figure 4:**
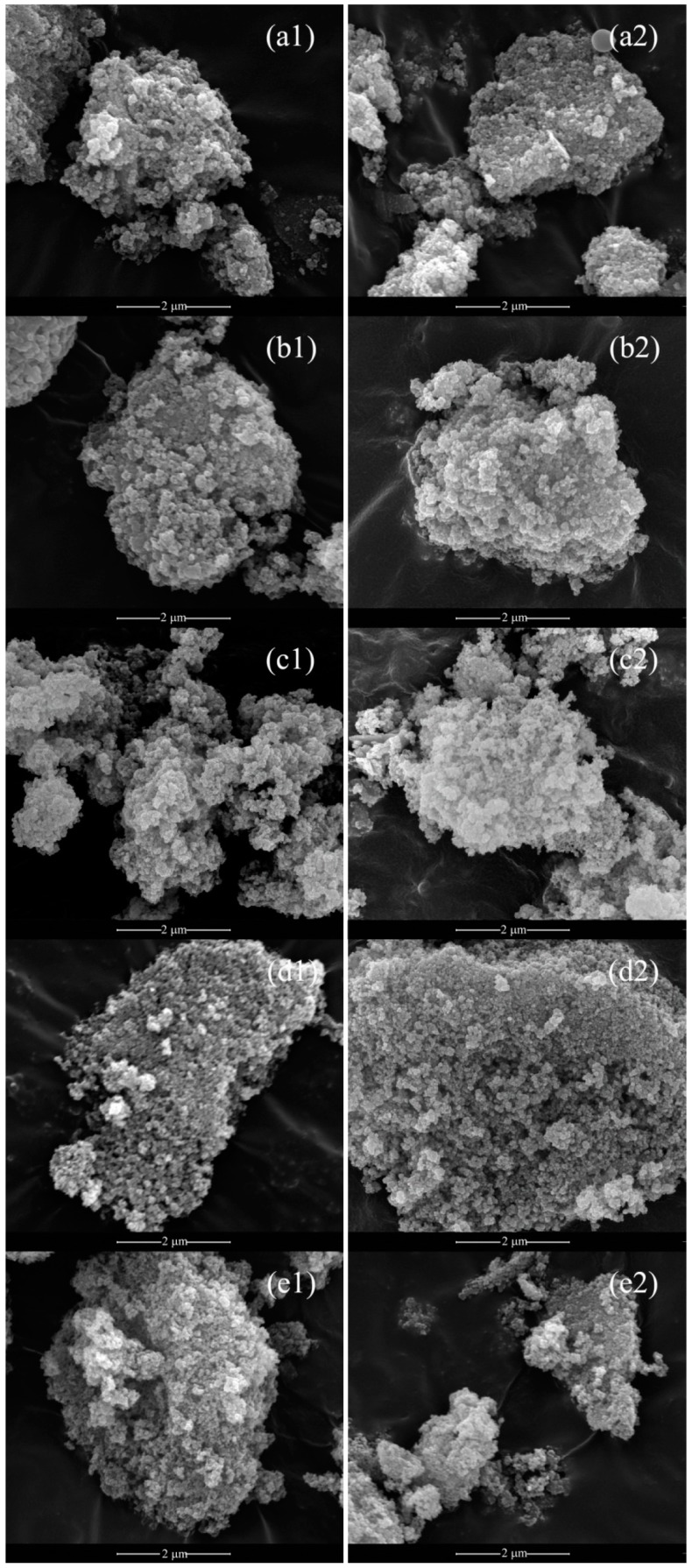
SEM micrographs at magnification of 50000X of as-synthesized (1) and calcined samples (2): a = C, b = GDC20, c = SDC20, d = GPrDC20, and e = SCaDC20.

The morphology of both the as-synthesized powders and the calcined ones is shown in Figure 4. One can notice (see Figure 4(a1),(a2) for sample C, and analogously for the other samples) the very similar microstructure for each sample between the as-synthesized and the calcined powders so confirming that in cerium-based carbonates, powder morphology is preserved upon the calcination step [4]. Furthermore, the microstructure is very similar among the various samples; in other words, irrespective of the actual composition of the product, the amorphous coprecipitates share the same morphology, i.e., the amorphous nature of the coprecipitates is a key point to establish such morphology. In more detail, this one is characterized by rounded clusters of spherical-like nanometric particles, with homogeneous size; in particular, after the calcination step, these clusters are indeed soft agglomerates of spherical-like particles of fluorite-type ceria, with the composition variable as a function of the samples, which size is in the order of some tenths of nm, which is well consistent with the crystal size reported in Table 2. These features are all valuable in view of forming and sintering processes.

**Figure 5:**
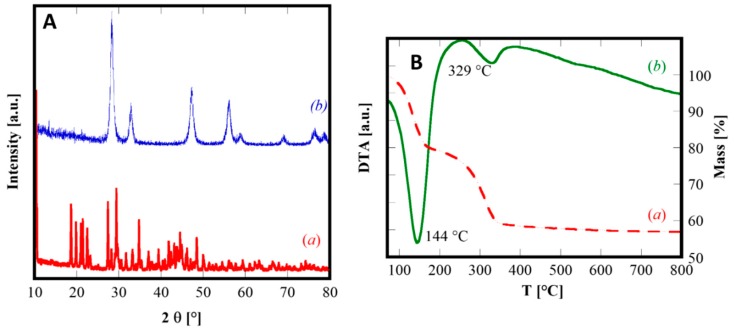
XRD patterns (**A**) of as-synthesized (a) and calcined (b) and DTA-TGA curves (**B**) of SDC20R10S sample.

Even though the obtained precipitates were all amorphous, it is well-known [30] that ceria-based coprecipitates show a strong tendency to crystallize even at room temperature. The adopted chemical-physical condition (very short duration of the process, i.e., fast mixing and low excess of precipitating agent) are specifically selected to avoid the crystallization of the as-prepared coprecipitates which could negatively influence the morphology of the powders. In fact, by changing the precipitation parameters, the results can be very different. To highlight that, a further precipitation experiment was carried out in the presence of a large excess of ammonium carbonate (R = 10) and by slow mixing of the solutions A and B. In these conditions, the nature of the precipitated powders is quite different. Figure 5A(a) shows the diffraction pattern of as-synthesized SDC20R10S sample, perfectly highlighting the crystalline nature of this material, in which all XRD peaks are attributable to cerium carbonate octahydrate, belonging to the group of lanthanites owning orthorhombic crystal structure (corresponding to the ICDD, International Centre for Diffraction Data, card n. 83-1211). Actually, the XRD pattern in Figure 5 exhibits a small peak shift, but it is perfectly justifiable, as the composition reported in the ICDD card and the actual composition are rather different, being (LaCe)(CO_3_)_3_·8H_2_O and (Sm_0.20_Ce_0.80_)_2_(CO_3_)_3_·8H_2_O, respectively. The total weight loss measured by DTA-TG analysis (reported in Figure 5B) is approximately 43%, perfectly agreeing with the theoretical value of 43.3%, further confirming the complete crystallization of the orthorhombic Sm-doped cerium carbonate octahydrate, during the coprecipitation at room temperature with slow mixing and with a large excess of carbonate ions. The DTA plot reveals two endothermic peaks, the first one at 144 °C related to water evolution and the second one at 329 °C representing the global effect of carbonate decomposition, Ce^3+^ oxidation, and crystallization of fluorite-type ceria. By calcination at 600 °C for 1.5 h, as predictable from the DTA plot, the sample SDC20R10S is completely crystallized in the fluorite-type structure (see Figure 5A(b)). Its crystallographic parameters are reported in Table 2 and, in particular, it can be noticed the nearly equal lattice parameter compared to sample SDC20 owning the same composition. 

**Figure 6:**
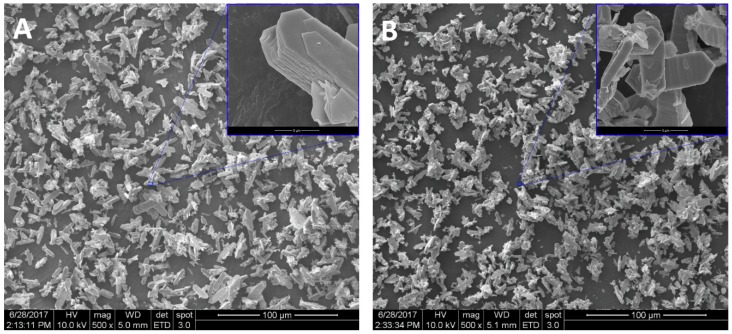
SEM micrographs (insets use higher magnification) of as-synthesized (**A**) and calcined (**B**) SDC20R10 samples.

Obviously, the powders morphology is dramatically influenced by their crystalline nature as evident from the SEM micrographs shown in Figure 6 of SDC20R10S, related to both as-precipitated and calcined powders. The orthorhombic Sm-doped cerium hydrated carbonate exhibits a very peculiar morphology, being constituted by elongated bidimensional, hexagonal-like particles. The largest size of those particles is in the range of 10 to 20 μm, whereas the smallest ones are of some μm; the average particles thickness, on the contrary, is of only some hundreds of nanometers (see inset in Figure 6A). As predictable, this peculiar morphology is preserved after the calcination step (see Figure 6B), with the particles of fluorite-type Sm-doped ceria sharing this same shape and therefore severely impairing the powders forming step. In fact, an efficient compaction of particles with such a shape is rather difficult and, consequently, the achieved green density is quite low, thus making a satisfying densification upon sintering hard. Furthermore, and perhaps more importantly, the size of crystallite calculated by the Scherrer formula applied on (111) peak, is around 14 nm, i.e., well lower than the size of particles shown in Figure 6B. Therefore, these particles are indeed aggregates of many and definitely smaller grains so that, very likely, these aggregates will behave as hard agglomerates during sintering, thus totally jeopardizing the pellets full densification.

**Figure 7:**
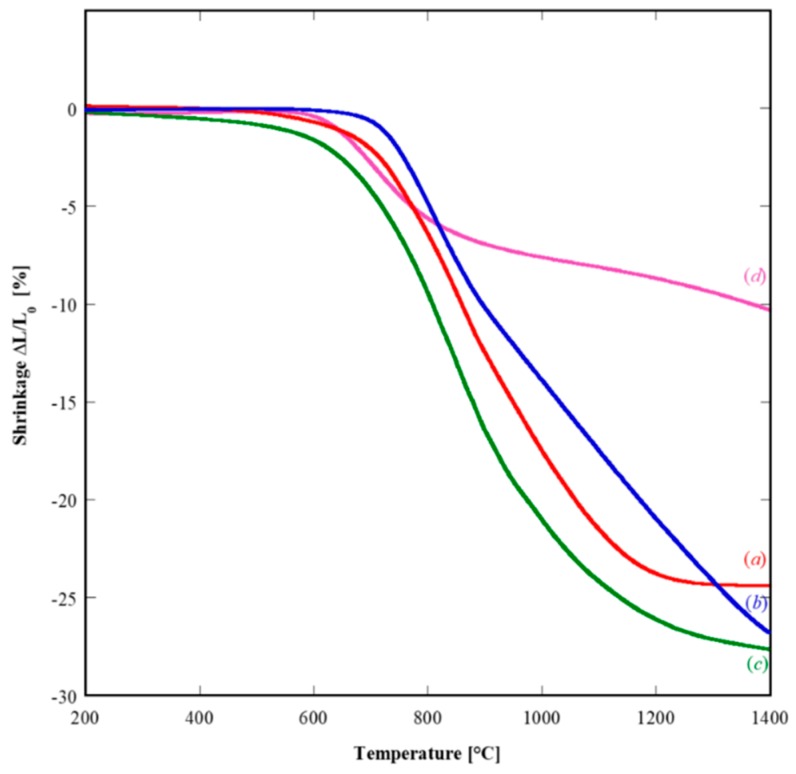
Thermodilatometric curves of samples SDC20 (green), GDC20 (blue), GPrDC20 (red), and SDC20R10S (fuchsia).

All the calcined powders were formed by uniaxial pressing at 1.2 ton for 60 s; their sintering behavior was studied by thermodilatometric analysis. Figure 7 displays the thermodilatometric plots of representative samples in which shrinkage, δL/L_0_, is plotted as a function of temperature.

The SDC20-related and GPrDC20-related (both amorphous precursor) dilatometric plots (see Figure 7(a),(c), respectively) exhibit the typical behavior of a highly sinterable material with only one densification step. In fact, the densification starts approximately at 650 °C with a fast increase of the shrinkage rate until its maximum value (−0.075%/°C and −0.060%/°C for GPrDC20 and SDC20, respectively) is reached at about 850 °C. Then the sintering rate slows down, becoming zero at ~1300 °C when the densification is practically ended with a total shrinkage equal to almost 28% for SDC20, and at 1200 °C for GPrDC20 with a total shrinkage of ~24%. The slightly better sintering behavior of GPrDC20 is very likely related to a better forming aptitude, i.e., a higher initial green density for this sample. The thermodilatometric plot of GDC20 sample, deriving again by an amorphous precursor (see Figure 7(b)), is analogous to the above described ones. Some slight difference appears, basically because its densification is still not completed at 1400 °C, although exhibiting a large shrinkage (~27%). On the contrary, the SDC20R10S-related dilatometric plot (crystalline precursor) is totally different. First of all, the total shrinkage at 1400 °C is only 10%. Furthermore, the maximum densification rate is reached at a very low temperature (700 °C) with a value about 0.035%/°C, i.e., roughly equal or lower than the half of maximum shrinkage rates of the other samples obtained from amorphous precursors. The most probable reason of this behavior is that the partial densification obtained at low temperature is related to sintering phenomena within the hard agglomerates (see Figure 6B). These phenomena did not concern spatial rearrangements of the agglomerates driven by mass transport, and consequently the global shrinkage was very low (~8%) when this “sintering” ended (~1000 °C). Upon a further increase of temperature, a very slow and progressive shrinkage of the sample, likely related to almost negligible spatial rearrangements of the agglomerates, occurred at a very low rate (i.e., lower than 0.01%/°C) up to 1400 °C when the dilatometric run was stopped. 

**Figure 8:**
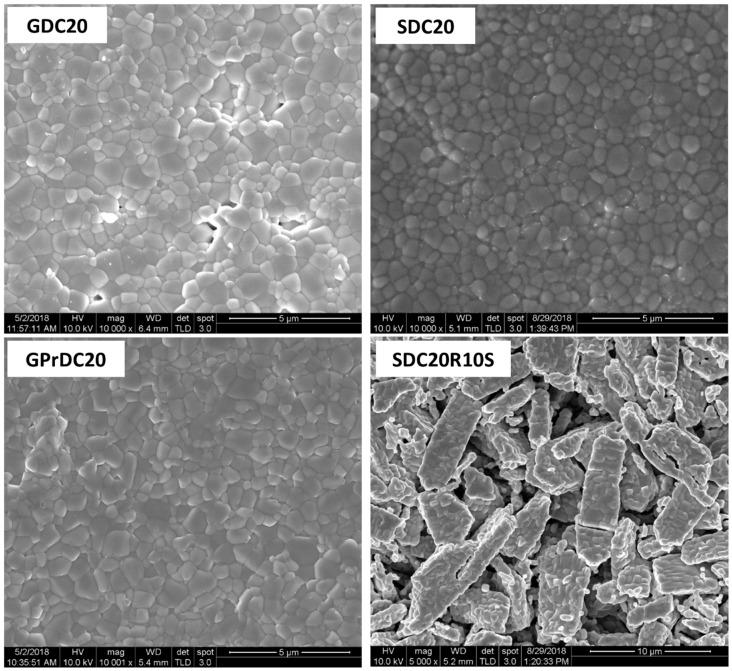
SEM micrographs of some sintered pellets: GDC20, SDC20, GPrDC20, and SDC20R10S.

Based on the thermodilatometric results, the sintering runs were carried out at 1300 °C for 3 h in air by using 5 °C/min as heating rate. The microstructure of the sintered pellets is displayed in Figure 8. All samples deriving from amorphous coprecipitates exhibit a homogeneous microstructure without any secondary phases. They are all well densified and, in particular, sample GPrDC20 is almost perfectly densified with virtual absence of any residual porosity and a grain structure characterized by homogenous and equiaxed grains whose size is ~1 μm with a narrow size distribution. As previously reported [31], the presence of even small Pr amount in fluorite-structured ceria enhances its final density, partly because of a higher green density induced by Pr as codopant. So, it is again confirmed in Figure 7, where the total shrinkage of GPrSDC20 about 24%, despite its final relative density is close to 100%, whereas for samples SDC20 and GDC20 the final shrinkage is in the range of 27 to 28% with a slight lower final relative density. Sintered samples GDC20 and SDC20 exhibit the same general microstructural features, even if a lower relative density has been achieved in this case. On the contrary, sample deriving from crystalline coprecipitate exhibits a very different microstructure (see Figure 8), characterized by the presence of bidimensional and perfectly densified primary particles, whose size is in the order of several micrometers, along with the presence of a diffuse and continuous porosity. These particles are very similar to the ones present in the calcined precursors (Figure 6), thus confirming the hypothesis formulated during the dilatometric plots discussion. Indeed, this microstructure is the one typically attributable to ceramics characterized by the presence of hard agglomerates formed upon calcination. Therefore, the crystallization of the as precipitated products greatly favors the formation of hard agglomerates in the corresponding calcined powders, thus making much more difficult or maybe even impossible, an adequate densification of those ceramics.

Finally, the electrical characterization of some selected samples was carried out in order to ascertain their aptitude as ceramic electrolyte for IT-SOFCs.

**Figure 9:**
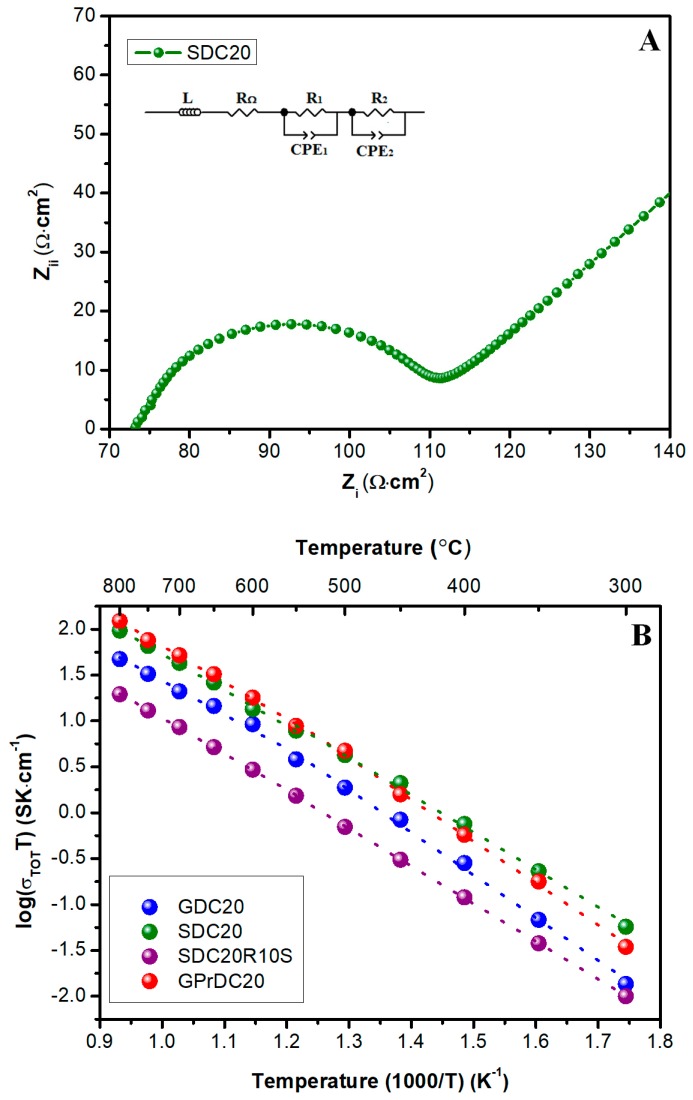
Nyquist plot for SDC20 at 500 °C (**A**) and Arrhenius plot of total conductivity for SDC20, GPrDC20, GDC20, and SDC20R10S (**B**).

The electrochemical data are fitted using an equivalent circuit to estimate the total resistivity values. This circuit consists in two (R-CPE, i.e., Constant Phase Element) elements joined together in series representing the grain boundary (R_gb_-CPE) and the electrode contribution (R_el_-CPE). In fact, from 800 °C to 550 °C the Nyquist plot showed a single semicircle due to electrode interface contribution, while at lower temperatures the total conductivity depends also by the grain boundary influence, as reported in Figure 9A at 500 °C for a representative sample.

This behavior is in agreement with literature [18,32]. The measured data reported as Arrhenius plots of the total conductivity are shown in Figure 9B where GPrDC20 exhibits the largest total ionic conductivity values in the temperature range of 500 to 800 °C. This feature is in agreement with the above-reported microstructural analysis, being these electrolytes denser, less porous and, thus, more conductive than the other samples. However, at lower temperatures, GPrDC20 total conductivity decreases quicker than SDC20, mainly because of their different grain boundary contribution. In all the electrolytes, a change in the linear slope is observed ~550 °C, the same temperature at which the grain boundary contribution becomes predominant in the total conductivity calculation. Therefore, two distinct values of activation energy at T > 550 °C and T < 550 °C can be detected as summarized in Table 3. 

## 4. Conclusions

A simple and cheap coprecipitation process carried out at room temperature using ammonium carbonate as precipitating agent is proposed to produce variously doped and codoped ceria-powders. This cycle proved to be reliable for obtaining amorphous cerium/carbonate systems and the very positive role of the amorphous nature of coprecipitates (formed by rare-earth hydroxycarbonates) has been highlighted in order to synthesize precursors for fluorite-structure ceria powders with excellent sintering aptitude. The molar ratio between carbonate ions and total metal cations, along with the effect of different mixing speed were found to greatly influence the as-formed phases and their morphology, allowing for control of powder properties and sintering behavior. In particular, it has been highlighted that amorphous precursors always lead to final fully dense materials, and the chemical/physical synthesis conditions to avoid ceria crystallization at room temperature are given. Furthermore, the proposed process is robust and works independently from the actual composition of the starting ceria-based system, provided that a solid solution of dopants/codopants fluorite-structured ceria can be formed. In addition, this work further provides evidences that some specific ceria codopants (i.e., Pr) can ease the densification process. Definitely, starting of powders prepared with the presented synthesis process, it is possible to produce ceria-based electrolytes with very high final densities and excellent ionic conductivities by adopting mild sintering cycles (i.e., low firing temperature and short durations). Finally, the consistent advantages in terms of simplicity, cost reductions, and high-value electrolytes properties make this process perfectly suitable for obtaining ceria-based ceramic electrolytes for IT-SOFCs, potentially even at industrial level.

## Figures and Tables

**Table 1:** Synthesized samples, composition, and coprecipitation conditions.

Sample	Composition	R/mix *
C	CeO_2_	2.5/F
GDC20	Gd_0.20_Ce_0.80_O_1.90_	2.5/F
SDC20	Sm_0.20_Ce_0.80_O_1.90_	2.5/F
GPrDC20	Gd_0.16_Pr_0.04_Ce_0.20_O_1.90_	2.5/F
SCaDC20	Sm_0.16_Ca_0.04_Ce_0.20_O_1.88_	2.5/F
SDC20R10S	Sm_0.20_Ce_0.80_O_1.90_	10/S

* R = molar carbonate anions/total metal cations ratio. F = fast mixing of precursors. S = slow mixing of precursors.

**Table 2:** Lattice parameter and crystallite size of calcined samples.

Sample	*a* (nm)	d_cryst_ (nm)
C	0.5411 ± 2.67 × 10^−4^	15
GDC20	0.5422 ± 1.36 × 10^−4^	12
SDC20	0.5433 ± 2.71 × 10^−4^	11
GPrDC20	0.5425 ± 4.22 × 10^−4^	11
SCaDC20	0.5431 ± 3.28 × 10^−4^	12
SDC20R10S	0.5435 ± 3.04 × 10^−4^	14

**Table 3:** Total conductivity and activation energies for sintered electrolytes.

	SDC20	GPrDC20	GDC20	SDC20R10S
300 °C	9.9 × 10^−5^	6.2 × 10^−5^	2.4 × 10^−5^	1.7 × 10^−5^
350 °C	3.7 × 10^−4^	3.6 × 10^−4^	1.1 × 10^−4^	6.1 × 10^−5^
400 °C	1.1 × 10^−3^	8.5 × 10^−4^	4.2 × 10^−4^	1.8 × 10^−4^
450 °C	2.9 × 10^−3^	2.2 × 10^−3^	1.2 × 10^−3^	4.2 × 10^−4^
500 °C	5.5 × 10^−3^	6.1 × 10^−3^	2.4 × 10^−3^	9.1 × 10^−4^
550 °C	9.5 × 10^−3^	1.1 × 10^−2^	4.6 × 10^−3^	1.8 × 10^−3^
600 °C	1.5 × 10^−2^	2.0 × 10^−2^	1.1 × 10^−2^	3.4 × 10^−3^
650 °C	2.8 × 10^−2^	3.5 × 10^−2^	1.6 × 10^−2^	5.6 × 10^−3^
700 °C	4.4 × 10^−2^	5.3 × 10^−2^	2.2 × 10^−2^	8.8 × 10^−3^
750 °C	6.4 × 10^−2^	7.4 × 10^−2^	3.2 × 10^−2^	1.3 × 10^−2^
800 °C	8.9 × 10^−2^	1.1 × 10^−1^	4.4 × 10^−2^	1.8 × 10^−2^
Ea (T > 550 °C) (eV)	0.77	0.79	0.73	0.77
Ea (T ≤ 550 °C) (eV)	0.81	0.88	0.91	0.81
